# Finite element comparative analysis of three different internal fixation methods in the treatment of Pauwels type III femoral neck fractures

**DOI:** 10.1186/s12891-022-06003-3

**Published:** 2022-11-29

**Authors:** Ji Ma, Ziying Zhao, Xiaodong Zhi, Hao Wang, Wei Wang

**Affiliations:** 1 Department of Orthopedics, First Affiliated Hospital of Jinzhou Medical University, Liaoning Province 121000 Jinzhou, China; 2 Department of Endocrinology, First Affiliated Hospital of Jinzhou Medical University, 121000 Jinzhou, Liaoning Province China; 3 Liaoning Key Laboratory of Medical Tissue Engineering, 121000 Jinzhou, Liaoning Province China; 4 Institute of Orthopedics, Jinzhou Medical University, 121000 Jinzhou, Liaoning Province China

**Keywords:** Femoral neck fracture, Pauwels III type, Finite element analysis, Biomechanics

## Abstract

**Background:**

Comparison of 4 cannulated lag screws (3 inverted triangular cannulated screws + anti-rotating screws;4 CLS), dynamic hip screws + derotational screws (DHS + DS), and femoral neck fixation system (FNS) in the treatment of Biomechanical properties of middle-aged Pauwels type III femoral neck fractures.

**Methods:**

The femur CT data of a healthy young volunteer was selected and imported into Mimics software to construct a three-dimensional model of a normal femur. Pauwels type III femoral neck fractures were simulated according to the 70° fracture line. Use Geomagic and SolidWorks software to optimize and build CLS, DHS + DS, and FNS fracture internal fixation models. Finally, Ansys software was used to analyze the stress distribution, peak value, and maximum displacement of the proximal fracture fragment and internal fixation; the displacement distribution, and peak value of the fracture surface at the fracture end.

**Results:**

① The stress peaks of the proximal fracture fragments in the three groups were concentrated near the femoral calcar. The peak stress of the FNS group was the largest, and the DHS + DS group was the smallest. ②The displacement of the fracture fragments was all located at the top of the femur. The peak displacement of the FNS group was the largest, and the DHS + DS group was the smallest. ③ The internal fixation stress of the three groups is concentrated in the middle part of the device. The stress distribution of the first two groups of models is more uniform than that of FNS. The peak stress of FNS is the largest and the CLS is the smallest. ④ The internal fixed displacements are all located at the top of the model. The peak displacement of the CLS is the largest, and the DHS + DS is the smallest. ⑤ The displacement of the fracture surface is in the upper part of the fractured end. The peak displacement of the FNS group was the largest, and the DHS + DS group was the smallest.

**Conclusion:**

Compared with the other two internal fixation methods, dynamic hip screw + derotational screw (DHS + DS) showed good biomechanical stability. When Pauwels type III femoral neck fracture occurs in young adults, DHS + DS can be given priority as the preferred treatment for this type of fracture.

## Introduction

More than 2 million hip fractures have been reported worldwide each year. It is expected that the number of cases will increase rapidly to 4.5 million worldwide by 2050, and the incidence of femoral neck fractures will also increase from 30–50% [1]. Femoral neck fractures in young adults are mostly caused by high-energy trauma such as traffic accidents and falling from height. Therefore, unstable Pauwels type III fracture caused by shear force is the main type of fracture. Based on the anatomical and biomechanical characteristics of the femoral neck, postoperative complications such as nonunion and osteonecrosis of the femoral head are prone to occur after fracture surgery, which further leads to high disability and high mortality [2]. Therefore, good anatomical reduction and solid internal fixation are the basis for the treatment of this type of fracture. Although the methods for the treatment of femoral neck fractures have been constantly improved and optimized, the incidence of postoperative complications has not been significantly improved, and the optimal surgical plan has not been unified [3]. Currently, dynamic hip screws (DHS) and cannulated lag screws (CLS) are the main surgical techniques widely used in clinical practice. Johnson et al. [4] found that, Although the triple inverted CLS is more effective in resisting torsion, it is not equal in resisting vertical shear and bending forces, which are considered to be the dominant deformation forces in unstable femoral neck fractures. To address this issue, researchers found that the placement of a transverse screw horizontally below the greater trochanter optimizes femoral mechanical stability [5]. Related studies have shown that [6]. If only DHS internal fixation is used, the resistance to rotation is even weaker than multiple CLS internal fixation. The anti-rotation screw (DS) inserted in parallel DHS can effectively enhance the anti-rotation ability of internal fixation [7, 8]. FNS is widely used in clinical practice because of its minimally invasive and stable advantages. For Pauwels type III fractures, there has been no biomechanical study comparing the biomechanical stability of CLS, DHS + DS, and FNS. This study aims to verify the mechanical stability of three internal fixation methods in the treatment of Pauwels type III femoral neck fractures by finite element analysis (FEA). It is hoped to provide some biomechanical basis for future clinical treatment.

## Materials and methods

A healthy adult male volunteer was selected. The height of the patient was 178CM and the weight was 70KG. The patient was in good health, with no history of surgery, trauma, osteoporosis, chronic diseases, or infectious diseases. The patients were aware of the purpose of the experimental study and signed the informed consent form. The study protocol was conducted by the relevant ethical requirements of the First Affiliated Hospital of Jinzhou Medical University. Siemens 128-slice spiral CT machine was provided by the First Affiliated Hospital of Jinzhou Medical University.

### Building a geometric model

Thin-slice CT scan was performed on the pelvis and lower limbs of the subjects. The image data of the patients were read and saved in Dicom format. The Dicom format was imported into Mimics21.0 software. The models were sequentially preprocessed by commands such as new mask, region growing, multilayer editing, intelligent fill, and smooth entity. After the model was transformed into a solid object, it was saved in STL format and imported into Geomagic Wrap 2021 software for processing. Refine the model with commands such as feature removal, nail removal, quick smoothing, carving, shell extraction, offset, fill, etc. The completed femur model was successively ordered to perform accurate surface, draw and construct contour line, construct the surface piece, construct a grid, and fit surface. Finally, the processed femoral model (cortical bone + cancellous bone) was saved in STP format and imported into SolidWorks 2022 software. The femoral cortical bone and cancellous bone models were combined by a Boolean operation to construct the final femoral model.

### Building the Pauwels III femoral neck fractures model

**Fig. 1:**
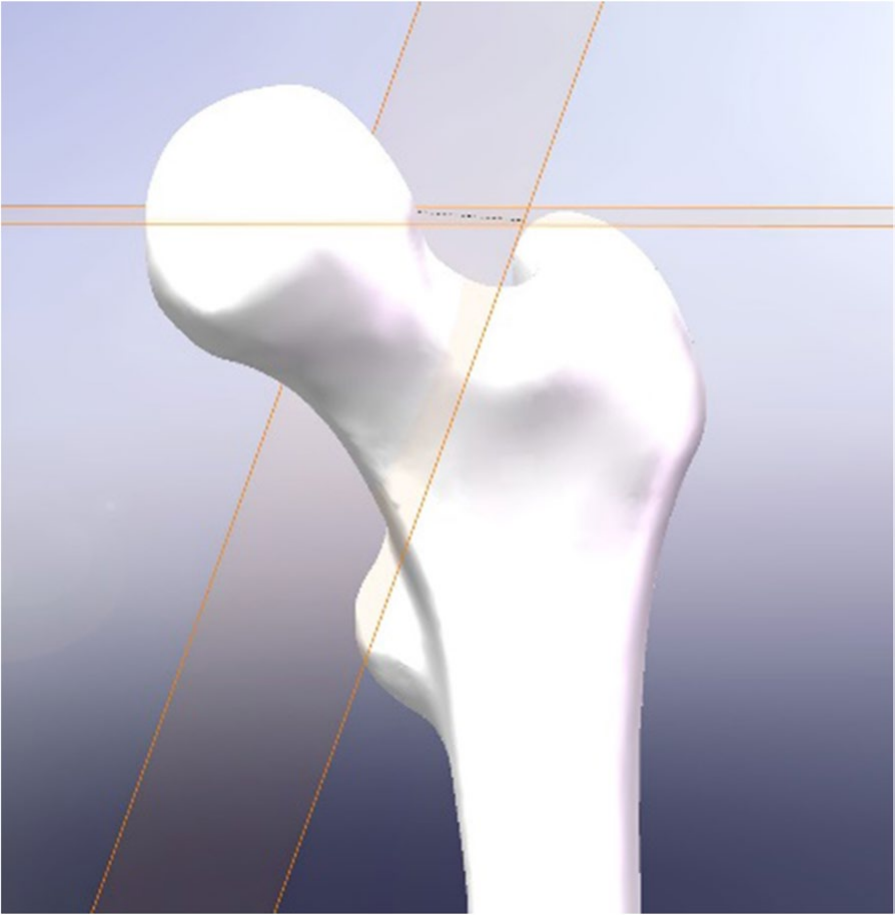
Model of Pauwels III femoral neck fracture

The processed final femur model was integrated into SolidWorks 2022 software. A datum was established through the center of the femoral head, and a second datum with an Angle of 70° to the plane was established through this datum. The second datum was used to segment the femoral model to simulate Pauwels type III femoral neck fracture in young adults. Model of Pauwels III femoral neck fracture was shown in Fig. 1.

### Building the internal fixation model

**Fig. 2:**
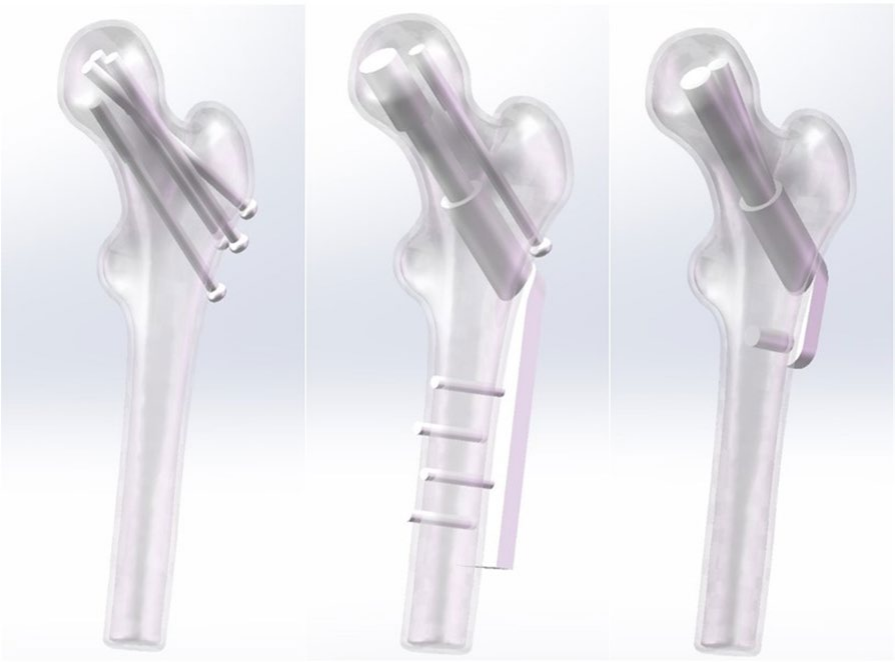
Schematic diagrams of the three internal fixation models. Figure note: from left to right in turn CLS, DHS + DS model, FNS model

Based on the current clinical treatment of femoral neck fractures with commonly used instruments and methods, three types of fracture internal fixation models were constructed by SolidWorks software. Since the focus of the experiments was not related to the threads, the threads were simplified to a solid cylindrical model. The internal fixation model was shown in Fig. 2.

### CLS model

The diameter of the smooth part of the screw was 4.8 mm, the diameter of the screw hollow was 2.5 mm, the diameter of the distal thread was 7.3 mm, and the length of the thread was 16 mm. The first three screws were placed parallel to each other in an inverted manner, and the last screw was placed slightly horizontally at the lateral femoral wall below the greater trochanter. The distal end of the four CLS was approximately 5 mm from the articular surface.

### DHS + DS model

The 4-hole DHS plate (135° nail plate Angle) was selected. The thickness of the plate was about 6.2 mm, and the length was about 70 mm. The lag screw was 90 mm in length, 8.2 mm in diameter, and the locking screw was 4.5 mm in diameter and 30-40 mm in length. Anti-rotation screw (DS) smooth screw diameter 4.8 mm, screw hollow diameter 2.5 mm, thread diameter 6.5 mm, thread length 16 mm. The DHS was close to the femur, the matching lag screw was close to the lower part of the femoral neck center, and the anti-rotation nail was placed parallel to the femoral neck above the matching lag screw. The distal end of both screws was approximately 5 mm from the articular surface.

### FNS model

The system is composed of three parts, namely a locking plate, bolt, and anti-rotation screw. The locking plate was a 130° angulation stabilization device, and the diameter of the locking hole screw was 5 mm. Bolt diameter 10 mm, round head, no thread, length 110 mm; The diameter of the anti-rotation screw is 6.4 mm, which can be locked with the bolt to increase the anti-rotation and improve the stability of the device. The FNS device was placed below the greater trochanter so that the bolt and anti-rotation screw was located in the center of the femoral neck, and the distal end of the bolt was about 5 mm from the articular surface.

### Material parameter setting

The experimental models were all ideal continuous, homogeneous, isotropic linear elastic materials, According to previous studies [9], The material parameters of the finite element model in this study are shown in Table 1.

**Table 1:** Material parameters of finite element models

Material Name	Elasticity modulus(MPa)	Poisson’s ratio
Femoral cortical bone	16,800	0.30
Femoral cancellous bone	840	0.29
Internal fixation (titanium alloy)	110,000	0.33

### Mesh generation

In this study, a 0.5-3 mm tetrahedral mesh was used for convergence testing. The results showed that the equivalent stress values calculated by 0.5-1 mm mesh were similar. Based on this, the mesh of the femur and internal fixator in this study was divided by 1.0 mm to generate mesh and unit. The number of nodes and elements of the three finite element models are shown in Table 2.

**Table 2:** Number of nodes and elements of three finite element models

Project	CLS	DHS + DS	FNS
Node number	482,352	523,773	486,040
Element number	312,588	338,304	314,087

### Constraints and load settings

The fracture site of the femoral neck was regarded as a complete fracture with good reduction, and the friction coefficient of the fracture end was set to 0.2. The contact area between the femur and the screw or plate was set as the binding state. The distal femur constrained the degrees of freedom. To truly model the force mode of the femur in daily life, according to previous literature reports [10], In this experiment, a coordinate system was established in the weight-bearing center region of the femoral head. The X-axis of the coordinate system was at an Angle of 13 degrees to the coronal plane of the femur, and at an Angle of 8 degrees to the sagittal plane. A load of 700 N along the X-axis is applied, thus simulating the bearing capacity of an adult walking normally with a single leg loading. For this experimental study, the specific role of each muscle group was not considered.

### Main outcome measures

The stress distribution, peak stress, and maximum displacement of the proximal femoral fracture block and internal fixation in three different internal fixation models were observed. Observe the displacement distribution of the fracture surface and the maximum displacement value of the fracture surface at the fracture end.

## Result

### Stress distribution and peak value of proximal femoral fracture fragment

**Fig. 3:**
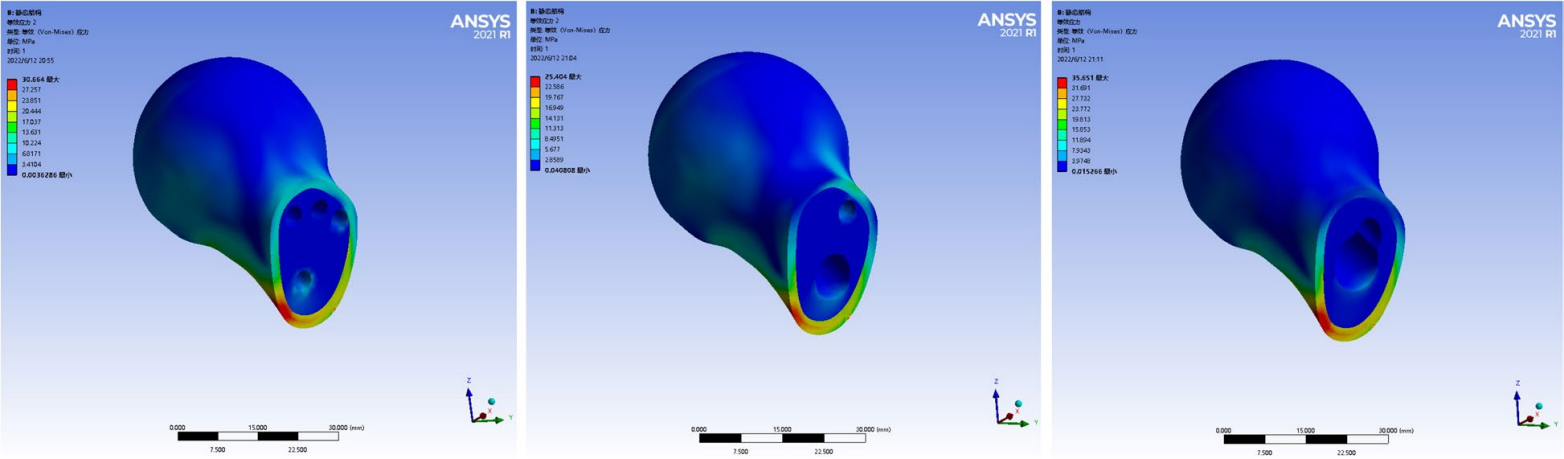
Stress distribution of the proximal fracture mass of the three internal fixation models. Figure note: From left to right, CLS models, DHS+DS models, and FNS models

The maximum stress of the proximal femoral fracture block in the three models was mainly concentrated on the medial and inferior side of the fracture line, close to the femoral callus. The peak stress of CLS models was 30.664 MPa. The peak stress of the DHS + DS model was 25.404 MPa. The peak stress of the FNS model was 35.651 MPa. The peak stress of the FNS group was the highest, and the peak stress of the DHS + DS group was the lowest, which was slightly smaller than that of the CLS group. As shown in Fig. 3.

### Displacement distribution and peak value of proximal femoral fracture fragment

**Fig. 4:**
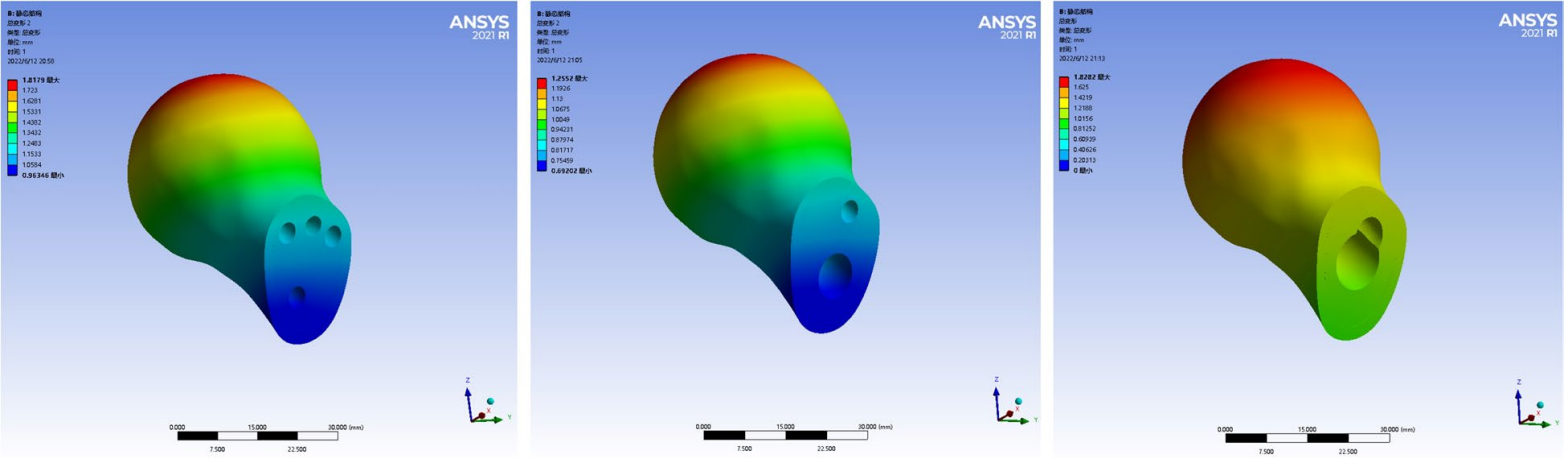
Deformation images of proximal fracture mass of the three internal fixation models

The maximum displacement of the proximal femoral fracture fragment in the three models was located at the upper part of the femoral head, and the displacement gradually decreased from near to far. The peak displacement of the CLS models was 1.817 mm. The peak displacement of the DHS + DS model was 1.255 mm. The peak displacement of the FNS model was 1.828 mm. The peak displacement of the FNS group was the largest, slightly larger than that of the CLS group, and the peak displacement of the DHS + DS group was the smallest. As shown in Fig. 4.

### Stress distribution and peak value of internal fixation

**Fig. 5:**
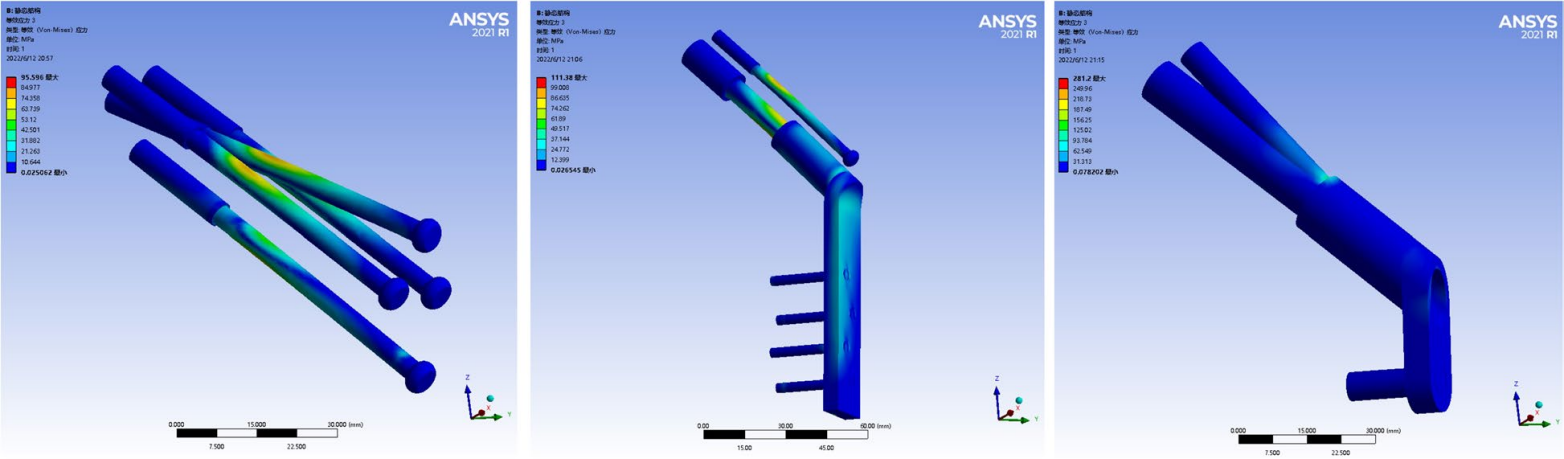
Stress distributions in the internal fixation devices used in the three models

The stress of the three internal fixation models was mainly concentrated in the middle part of the internal fixation device corresponding to the fracture line. The stress distribution of the first two internal fixation models was more uniform than that of the FNS. The stress magnitude gradually decreased from both ends of the middle image. The peak stress of CLS was 95.596 MPa. The peak stress of DHS + DS was 111.38 MPa. The peak stress of the FNS was 281.2 MPa. The peak stress of the FNS group was the largest, and the peak stress of CLS group was the smallest, which was slightly smaller than that of the DHS + DS group. As shown in Fig. 5.

### Displacement distribution and peak value of internal fixation

**Fig. 6:**
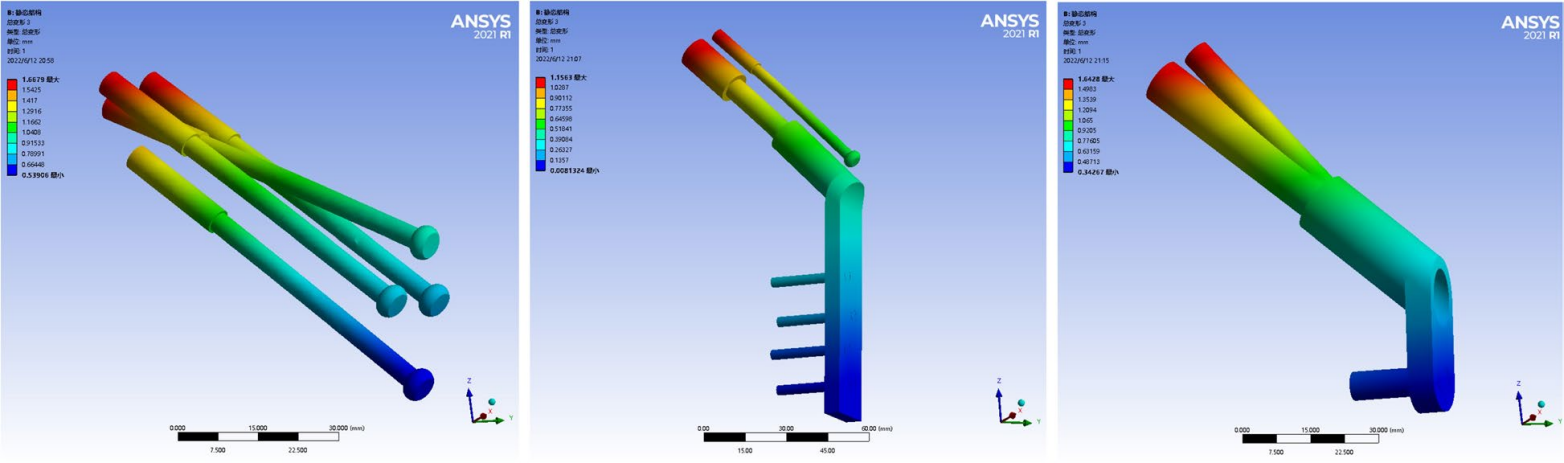
Deformation images in the internal fixation devices of the three models

The displacement of the three internal fixation models was mainly concentrated at the proximal end of the device. The maximum displacements were located at the very top of the internal fixation model with uniform distribution. The peak displacement of the CLS model was 1.667 mm. The peak displacement of the DHS + DS model was 1.156 mm. The peak displacement of the FNS model was 1.642 mm. The CLS group had the largest peak displacement, which was slightly larger than the FNS group, and the DHS + DS group had the lowest peak displacement. As shown in Fig. 6.

### Displacement distribution and peak value of fracture surface at fracture end

**Fig. 7:**
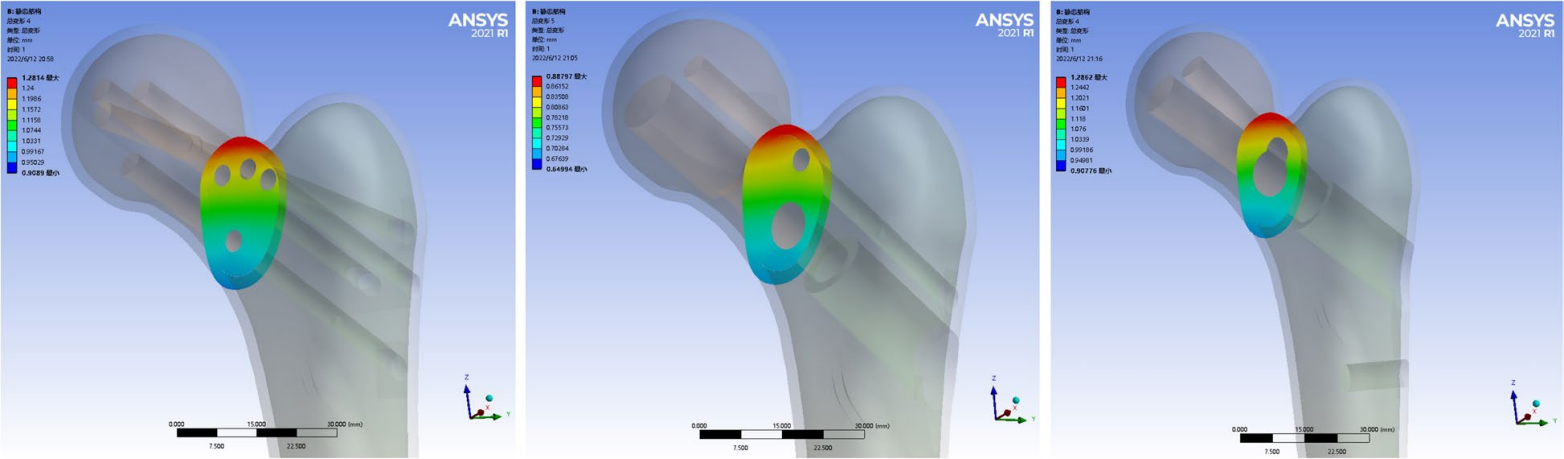
Deformation images in the fracture surface of the three models

The displacement of the fracture surface of the three internal fixation models was mainly concentrated in the upper part of the fracture end, which gradually decreased from near to far and was uniformly distributed. The peak displacement of CLS group was 1.281 mm. The peak displacement of the DHS + DS group was 0.887 mm. The peak displacement of the FNS group was 1.286 mm. The peak displacement of the FNS group was the largest, slightly larger than that of the CLS group, and the peak displacement of the DHS + DS group was the smallest. As shown in Fig. 7. Table 3 shows the summary results of the finite element analysis of the three internal fixation models.

**Table 3:** Finite element analysis results of three internal fixation models

Parameter	CLS	DHS + DS	FNS
Stress of the proximal fracture fragment (MPa)	30.664	25.404	35.651
Displacement of the proximal fragment (mm)	1.817	1.255	1.828
Internal fixation stress (MPa)	95.596	111.380	281.2
Displacement of internal fixation (mm)	1.667	1.1563	1.642
Displacement of fracture surface at the broken end (mm)	1.281	0.887	1.286

## Discussion

In recent years, with the development of computer technology, the finite element has become a common tool for biomechanical analysis. The finite element model can be used to evaluate the mechanical properties of fracture internal fixation through mechanical simulation. It enables the assessment of stress and strain generated within the bone prior to implantation of internal fixators. The finite element method is faster and yields more detailed data than traditional mechanical tests. In addition, biomechanical finite element models can be analyzed multiple times without ethical violations. According to the horizontal tilt Angle of the fracture line, femoral neck fractures were divided into 3 types by Pauwels. Pauwels type i fracture was defined as an Angle between the fracture line and the horizontal line less than 30°. Pauwels type ii fracture was defined as an Angle between 30 ° and 50°. Pauwels type III fracture was defined as an Angle greater than 50°. Pauwels type III fractures mostly occur in young and middle-aged people and are mostly caused by high-energy trauma. Relevant studies suggest that [11], Fracture line Angle can affect the stability and clinical prognosis of a femoral neck fracture. The larger the Angle of the fracture line, the greater the shear force on the fracture end, and the more unstable the fracture. This will lead to fracture malunion, avascular necrosis of the femoral head, nonunion and other complications. Although relevant studies such as biomechanical, controlled clinical experiments have been conducted to determine the most appropriate fixation method in the surgical treatment of unstable femoral neck fractures. There is still no consensus on the choice of the most ideal internal fixation device.

At present, the mainstream surgical method for unstable femoral neck fractures in young adults is multiple CLS and dynamic hip screw (DHS) fixation [12]. CLS have been the standard fixation method for femoral neck fractures in young patients for many years due to their advantages of less trauma, shorter operation time, and adequate fixation [13]. However, biomechanical studies have shown that [4], multiple CLS are more effective in providing anti-torsion stability and lower risk of femoral head blood supply injury. However, it can not well solve the risk of internal fixation failure caused by vertical shear and bending forces. Vertical shear force and bending force are considered to be the dominant deformation forces in unstable femoral neck fractures. Due to the poor mechanical stability of such fractures in the vertical position, varus deformity, nonunion, and osteonecrosis of the femoral head is often encountered after surgery. The literature has similarly reported 20–48% adverse outcomes after fixation of femoral neck fractures with three parallel hollow screws [14]. Although there is no consensus in the literature on the contribution of the fourth screw to the stability of internal fixation of femoral neck fractures. But there are also some studies that show increased stability with the use of a fourth screw [15]. Kauffman et al. [16] reported that when posterior femoral neck fractures were comminuted, fixation with four screws provided better stability than fixation with three screws. Gumustas et al. [5] compared the efficacy of CLS with three different configurations in the treatment of unstable femoral neck fractures from the perspective of biomechanics. They found that the use of a transverse screw in addition to the screw placed parallel to the femoral neck seemed to provide better stability in the treatment of unstable femoral neck fractures. The stability of using 4 screws is better than that of using 3 screws. However, Liporace et al. [17] evaluated 76 patients with Pauwels type III femoral neck fractures after 12 years of postoperative follow-up. Postoperative complications such as osteonecrosis and nonunion of the fracture occurred in 32% of patients treated with CLS and were lowest (14%) when DHS was chosen. Therefore, internal fixation devices with fixed angles, such as DHS, have been recommended for the treatment of Pauwels type III femoral neck fractures.

In vertically displaced fractures, shear and rotational forces can cause swing and rotation of the femoral head, which may lead to many postoperative complications such as failure of internal fixation, varus deformity, nonunion, and avascular necrosis of the femoral head [18]. Therefore, any fixation method needs to resist these forces during bone healing. Biomechanical and clinical studies have shown a slight improvement in shear resistance with DHS [19, 20]. However, because only a single screw is used, DHS is less able to resist rotational forces than when multiple CLS are used [21]. Therefore, patients treated with DHS are more likely to experience the risk of rotational displacement of the femoral head after surgery. In order to overcome this disadvantage, a surgical procedure has been developed to improve it by placing an anti-rotation screw (DHS + DS) in parallel above the DHS. Freitas et al. [22]analyzed the mechanical stability of DHS, DHS + DS, and three CLS in Pauwels type III femoral neck fractures by finite element modeling. The results of this study showed that the displacement and stress peaks of the DHS plus DS fixation device were smaller than those of the previous two groups when compared with DHS and CLS alone. The study by Sağlam et al. [23] similarly stated that when CLS were compared with DHS systems, DHS with additional anti-rotation screws performed better in terms of biomechanics. The results of in vitro biomechanical experiments by Samsami et al. [24] showed that the axial stiffness of the DHS + CS group was 404.3 N/mm, while the axial stiffness of the 3 CLS group was 243.1 N/mm. The above studies together showed that the combination of DHS + CS significantly improved the anti-shear and anti-rotation effects.

Several scholars have reported fixation methods of anatomic locking plates for unstable fractures of the femoral neck. Clinical studies and finite element analyses have also collectively shown that plates are superior to screws in terms of biomechanical stability [25]. Despite the many advantages shown in these studies, these surgical methods undoubtedly impair the blood supply to the surrounding tissues and femoral head. In order to solve this problem, the femoral neck internal fixation system (FNS), which is currently used in clinical practice, was developed. The femoral head is fixed in a minimally invasive way using a combination of locking anti-rotation screws and power bolts. The anti-rotation screw is locked in the bolt, allowing the two screws to slide together along the side plate for dynamic fixation. However, there are still different opinions on the clinical effect and biomechanical stability of the FNS in the treatment of femoral neck fractures. Relevant clinical studies have shown that FNS shows good clinical efficacy and biomechanical stability for unstable femoral neck fractures [26, 27]. Ma et al. [28] found that FNS had good biomechanical properties through clinical studies. The incidence of femoral neck shortening and screw cutting-out was lower than that of CLS group, but there was no significant difference in the probability of postoperative femoral head necrosis and nonunion. And they also found no significant difference between the FNS and DHS systems. FNS can provide a similar effect as DHS, achieving robust and stable fixation. However, the results of Xia et al. [29] found that the structural stability of FNS was weaker than that of CLS, CLS + medial plate, and biplanar support screws, suggesting that this method may not be as stable as previously reported. The results of this study were the same as those of the appeal study, and it was found that the peak stress of the proximal fracture block and internal fixation of the FNS were greater than those of the former two fixation methods. The displacement of the proximal fracture fragment and fracture surface was also slightly greater than that in the CLS group. The reason for this result may be due to the structure of the device itself. Because the device is not a single unit, there is a connection gap between the bolt and the anti-rotation screw, which will inevitably concentrate the stress at the connection of the device when the axial load is applied.

In this study, three kinds of internal fixation models of Pauwels type III femoral neck fractures were established by the finite element analysis method, and their biomechanical properties were compared and analyzed. The results of this experimental study showed that all four CLS and DHS + DS showed good biomechanical stability. The stress of the proximal femoral fracture block in the three models was mainly distributed near the femoral callus, and the peak stress and displacement of the fracture block in the DHS + DS group were lower than those in the CLS group. This suggests that this internal fixation counteracts most of the shear force when DHS + DS is used to fix unstable femoral neck fractures. The stress of the three internal fixation devices was concentrated in the middle region of internal fixation corresponding to the fracture end. Compared with the stress on the proximal femoral fracture fragment, the internal fixation carries more stress. In the three groups of internal fixation, the peak stress and displacement of FNS were greater than those of DHS + DS and CLS groups, indicating that the mechanical stability of the former two internal fixation methods was stronger than that of FNS. The stress of internal fixation was the lowest in the 4 CLS group, and the displacement of internal fixation device was the lowest in the DHS + DS group. The reason may be that the fourth transverse screw dispersed part of the shear vertical stress, which made the stress distribution of the screw more uniform and dispersed. The displacement of the fracture surface of the broken end showed that the displacement distance of DHS + DS was the smallest, and there was no significant difference between CLS and the FNS. Therefore, comprehensive analysis of the characteristics of various aspects, dynamic hip screws + anti-rotation nail (DHS + DS) can provide more stable stability, can provide a better biomechanical environment for fracture healing, further promote bone formation, and shorten the healing time.

There are some limitations in this study: ① The material parameter of the model is set as an isotropic elastic material, which will be different from the anisotropic material properties of the actual human skeleton. However, the purpose of this study was to model the trend as a whole. So such a setup can be considered reasonable. ② The fracture types and internal fixation devices were limited to a specific study population, and the experimental results did not support other fracture types and internal fixation methods. ③ This experiment is only conducted on the finite element method, and further experimental research on cadaver biomechanics is still needed for verification. ④ The load setting only simulates the carrying capacity under the condition of a single leg bearing weight during normal walking of adults, and the specific role of each muscle group is not considered. In the following research, the influence of the muscles around the hip joint on the femur should be considered.

To evaluate the biomechanical properties of three different internal fixation methods in the treatment of Pauwels type III femoral neck fractures in young adults. Although the peak stress of the four CLS internal fixation is the smallest, the stress and displacement peak of the proximal femoral fracture block are larger than those of the dynamic hip screws + anti-rotation nail (DHS + DS) model, just like the femoral neck system. When a femoral neck fracture occurs, it may not be conducive to bone growth. The biomechanical stability of DHS + DS is better than that of CLS and FNS model, which provides a good healing environment for fracture healing. Therefore, when Pauwels type III femoral neck fracture occurs in young adults, DHS + DS can be considered the preferred treatment for this type of fracture.

## Data Availability

The datasets generated and analyzed during the current study are not publicly available due to the data also forms part of an ongoing study but are available from the corresponding author on reasonable request.
